# Causal Mediation Analysis of the Effects of Pain Education on Disability and Pain Intensity in Individuals with Chronic Low Back Pain

**DOI:** 10.3390/jcm15010348

**Published:** 2026-01-02

**Authors:** Ahmed Alalawi

**Affiliations:** Department of Medical Rehabilitation Sciences, Faculty of Applied Medical Sciences, Umm Al-Qura University, Makkah 24382, Saudi Arabia; amalawi@uqu.edu.sa

**Keywords:** chronic low back pain, pain education, psychological well-being, self-efficacy, mediation analysis

## Abstract

**Background/Objective:** The purpose of this study was to determine whether the effects of pain education combined with physiotherapy could be explained by changes in psychological well-being and self-efficacy in individuals with chronic low back pain (LBP). **Methods:** This study includes a secondary analysis (mediation analysis) of a randomized controlled trial (RCT) that compares the effect of physiotherapy and pain education with physiotherapy alone. The Roland-Morris Disability Questionnaire, assessed at six weeks, was used as a primary outcome in this study, with pain intensity as a secondary outcome. The World Health Organization Five Well-Being Index (WHO-5) and the General Self-Efficacy Scale were evaluated as potential mediators. Causal mediation analysis based on a counterfactual framework was employed to estimate both direct and indirect effects. **Results:** The analyses comprised 46 participants (mean age = 42.2 years; 54.3% female) who received pain education along with physiotherapy. In the mediation models, improvements in emotional well-being (assessed by WHO-5) explained approximately one quarter of the effect of the intervention on disability (average causal mediation effect = −1.66, 95% CI [−2.8, −0.72], *p* < 0.001). By contrast, self-efficacy did not significantly mediate disability, and neither factor accounted for changes in pain intensity. Sensitivity analyses suggested that the indirect effect on psychological well-being was reasonably robust against potential unmeasured confounding factors. **Conclusions:** Enhancements in psychological well-being were associated with reductions in disability following pain education, whereas self-efficacy did not emerge as a significant mediator. These findings may support the value of incorporating mental well-being strategies within rehabilitation programs for chronic LBP.

## 1. Introduction

One of the leading causes of disability worldwide and a significant public health concern is chronic low back pain (LBP). According to estimates from around the world, LBP is the leading cause of years spent disabled in many different areas and demographic groups. Over the coming decades, it is anticipated that the number of people who suffer from LBP will keep rising [1]. High healthcare expenses, lost productivity, and a reduced quality of life are just a few of the significant personal, social, and financial costs associated with LBP [2].

According to current treatment guidelines, the first-line treatment for LBP should involve non-invasive techniques such as physiotherapy, exercise therapy, and patient education [3]. These interventions result in modest improvements in pain and function, but recurrence rates are still high, and many patients experience long-term disability [4]. This highlights how important it is to fully understand how rehabilitation interventions work in order to improve outcomes.

A biopsychosocial model recognizes that physical impairments, in addition to psychological and social factors, have an impact on pain and disability [5]. There is a consistent correlation between outcomes in chronic LBP and psychological measures such as self-efficacy, catastrophizing, and fear-avoidance [6]. Specifically, self-efficacy has been demonstrated to predict disability levels and quality of life in musculoskeletal pain conditions [7,8]. More general signs of mental health, mood, and resilience have also been linked to pain-related outcomes, and they often have a stronger link to disability than to pain severity itself [9]. Within biopsychosocial frameworks such as the fear-avoidance model [10] and the psychological flexibility model [11], cognitive and emotional processes play a central role in shaping pain-related disability. Both self-efficacy and psychological well-being are conceptually aligned with these frameworks, making them theoretically relevant mediators of rehabilitation outcomes. Unlike constructs such as catastrophizing or affective distress, psychological well-being reflects positive mental health and resilience rather than negative symptom burden [12] and has been relatively understudied as a mediator in LBP rehabilitation. Psychological responses to pain, including factors such as well-being and self-efficacy, can vary across age groups [13]. A recent umbrella review in individuals with chronic LBP reported that pain neuroscience education, when combined with exercise or physiotherapy, leads to improvements in pain and disability. However, the review also highlighted substantial heterogeneity and noted that the mechanisms underlying these benefits remain preliminary and require further research [14].

Despite this evidence, there exist relatively few randomized controlled trials (RCTs) that have systematically investigated the psychological mechanisms by which rehabilitation interventions confer their benefits. Numerous secondary analyses and mediation studies indicate that psychosocial alterations could explain a portion of the effects of interventions such as cognitive behavioral therapy (CBT), cognitive functional therapy (CFT), and exposure-based methodologies [15,16,17,18]. The Back Skills Training trial illustrated that modifications in cognitive and affective processes partially mediated enhancements in disability [18]. Concurrently, mediation analyses in exposure-based treatments identified significant pathways of change, including reductions in catastrophizing and fear-avoidance, alongside increases in self-efficacy [15]. Mindfulness-based stress reduction (MBSR) and CBT have demonstrated efficacy in alleviating pain and enhancing psychological and functional outcomes, partially through the enhancement of mindfulness, acceptance, and self-efficacy [19,20]. Recent mediation studies on physiotherapy, yoga, and chiropractic care have yielded inconclusive results, indicating that certain psychological factors, such as stress and self-efficacy, serve as partial mediators, whereas others, such as catastrophizing or sleep disturbances, do not [16,17,21,22].

This expanding body of literature highlights the necessity to identify the psychological mechanisms that most significantly impact clinical outcomes and to determine whether these mechanisms differ by intervention type. Nevertheless, significant gaps persist, as the majority of trials conducted thus far have excluded psychological well-being as a mediator, despite evidence correlating it with pain-related disability. Despite extensive mediation research in chronic LBP, the role of psychological well-being in pain education interventions remains largely unexamined, creating a conceptual gap that this study addresses. We hypothesized that improvements in psychological well-being would partially mediate the effect of pain education on disability. This study aimed to assess whether the impact of a pain education intervention on disability and pain intensity in individuals with chronic LBP was mediated by changes in self-efficacy, psychological well-being, and pain intensity itself. In line with this aim, the present work focuses on the effects of this intervention by applying a counterfactual causal mediation framework.

## 2. Materials and Methods

### 2.1. Study Design

The data from a previously published RCT that evaluated the effects of a structured pain education and physiotherapy program on people with chronic LBP were secondary analyzed in this study. Details about the study methodology, participants’ details, recruitment, and key findings are published elsewhere [23]. Briefly, a two-arm parallel-group design was used in the trial, and participants were randomized 1:1 to receive either standard physiotherapy or physiotherapy plus a pain education intervention. Randomization was performed using block randomization (block size = 4), with allocation sequences concealed in sealed, opaque envelopes to ensure allocation concealment. Participants were blinded to their group assignment, as were the physiotherapists who conducted the clinical outcome examinations. This study was conducted in accordance with the Declaration of Helsinki and reported in accordance with the Guidelines for Reporting Mediation Analysis [24]. Ethical approval was granted by the Institutional Ethics Committee (RP/114/2021), and the trial was registered with the Clinical Trial Registry (CTRI/2021/08/035963).

### 2.2. Participants

Until a sufficient sample size of 92 was reached, participants were recruited on a rolling basis [23]. Adults of either sex, ages 18 to 60, who had been diagnosed with nonspecific chronic LBP that had persisted for longer than three months were eligible. A medical professional used appropriate diagnostic investigations and clinical assessment to establish the diagnosis. Participants who missed more than two scheduled sessions or voluntarily withdrew were excluded from the study. Group assignment was concealed from both participants and outcome assessors. Prior to enrollment, all participants provided written informed consent.

### 2.3. Interventions

#### 2.3.1. Physiotherapy

A standardized physiotherapy program for chronic LBP was provided to both groups, based on international clinical practice guidelines [3,25]. The program included advice to avoid prolonged bed rest, application of superficial heat for 10 min, and stretching of the lumbar musculature for 10 min. It also comprised static cycling for 10 min and core stabilization exercises aimed at enhancing strength and endurance for 10–15 min. A study calendar was used to monitor adherence during the roughly 40 min sessions.

#### 2.3.2. Pain Education

Participants in the intervention group received a structured pain education program in addition to a regular physiotherapy program. The education intervention session covered topics like the nature of chronic pain, how pain affects the brain, how central sensitization works, what fear-avoidance beliefs are, and how social and psychological factors affect how pain is perceived. For individual education sessions, PowerPoint presentations, pictures, lectures, and interactive discussions were used. There were two sessions per week for the first three weeks. Reflective Q&A sessions took place in weeks four and five. Participants were given a printed pain education handbook at the end of the six-week program.

Following Butler and Moseley’s methodological framework, a culturally specific pain education handbook was developed specifically for this study [26,27,28]. Relevant clinical guidelines for LBP and the body of existing literature on pain education were used as the basis for the content. Additionally, the Explain Pain handbook [28] was used. The drafts of the education handbook were reviewed by several co-authors to ensure clarity, and final proofreading was performed by a physiotherapist, a clinical psychologist, a rehabilitation doctor, and a native speaker.

### 2.4. Measurement

#### 2.4.1. Outcome Measures

The disability and pain intensity were the primary outcomes of this mediation analysis. Disability was assessed using the Roland-Morris Disability Questionnaire (RMDQ), which consists of 24 items with a score range of 0 to 24 [29]. Higher disability is indicated by higher scores. The reliability and validity of the RMDQ have been demonstrated [29]. Pain intensity during routine tasks was measured using a 10-point Visual Analog Scale (VAS; 0 = no pain, 10 = worst imaginable pain). In clinical and research settings, the VAS is a valid and reliable measure for assessing pain intensity [30]. Scores of both measures were collected at baseline and at the end of the 6-week intervention [23].

#### 2.4.2. Mediators

Self-efficacy and psychological well-being were used as mediators in this study. Self-efficacy was assessed using the General Self-Efficacy Scale (GSE) [31]. This 10-item self-report test assesses a person’s level of confidence in their capacity to manage and succeed in challenging circumstances. Higher GSE scores indicate that people believe they are more self-sufficient. The scores range from 10 to 40. The construct validity and internal consistency have been demonstrated in a range of populations [31].

Psychological well-being was assessed using the World Health Organization Five Well-Being Index (WHO-5) [32]. It is composed of five questions that evaluate the current emotional state of participants. A raw score ranging from 0 (the lowest level of well-being) to 25 (the highest level). Additionally, a percentage score between 0 and 100 is calculated by multiplying the raw scores by 4, where higher values indicate greater well-being. In both clinical and non-clinical populations, the WHO-5 is known to have robust psychometric characteristics, including validity, reliability, and sensitivity to change [12]. Since psychological well-being and self-efficacy are key elements in biopsychosocial models of chronic pain and have both been shown to have an effect on pain-related outcomes and disability in people with chronic LBP, these mediators were chosen [33]. Pain intensity was included as a potential mediator because prior mediation research shows that pain contributes to disability indirectly by influencing cognitive and emotional processes such as fear of movement, catastrophizing, and self-efficacy [34].

### 2.5. Analysis

The proposed causal framework was designed to divide the total effect of the intervention into direct and indirect components, as previously employed [16]. A causal mediation analysis was conducted utilizing a counterfactual-based methodology to estimate the natural direct effect (NDE) and the average causal mediation effect (ACME), or natural indirect effect [35]. Within this framework, the NDE signifies the impact of the intervention on the outcome that remains unexplained by the mediator, whereas the ACME quantifies the degree to which the intervention affects the outcome via alterations in the mediator. The proportion mediated was determined by the ratio of the ACME to the total effect, indicating the extent to which the mediator pathway accounts for the intervention’s effect [16].

The analyses relied on the assumption of sequential ignorability: namely, no unmeasured confounding between intervention and mediator, between mediator and outcome, and no mediator–outcome confounders affected by the intervention [16]. Given the randomized design, confounding between intervention and mediator was not expected. Potential mediator–outcome confounders considered in the models included baseline age, sex, pain intensity, disability, and baseline values of the mediator.

To further assess robustness, additional sensitivity mediation models were estimated that included baseline smoking status, BMI, and duration of low back pain as covariates in both the mediator and outcome equations. These extended models yielded ACME, average direct effect (ADE), and total effect estimates that were nearly identical to the primary models, indicating that these baseline variables did not confound the mediation pathway.

The original RCT reported three missing baseline item responses (two items from the GSE and one item from the WHO-5), which were addressed using mean substitution at the item level, as described elsewhere [23]. Following this minimal item-level imputation conducted in the primary trial, the dataset used for the present secondary analysis contained no missing mediator, outcome, or covariate values at either baseline or follow-up. Therefore, no additional imputation procedures were performed, and the current mediation analysis was conducted on a complete-case dataset.

Although baseline mediator and outcome values were included as covariates, the mediator and outcome values used in the mediation pathway were both assessed at the 6-week follow-up. Baseline adjustment reduces confounding but does not establish temporal ordering. Consequently, the mediation findings should be interpreted as exploratory. To address assumptions underlying causal mediation, particularly the sequential ignorability assumption, we conducted a sensitivity analysis following the recommendations of Imai et al. (2010), quantifying the degree of unmeasured mediator–outcome confounding required to nullify the ACME [35].

Although baseline mediator and outcome values were collected and statistically adjusted for, mediator and outcome assessments were obtained at the same 6-week follow-up.

Separate models were estimated for each hypothesized mediator (self-efficacy, psychological well-being, pain intensity, and disability) and each outcome (pain intensity and disability). Following the approach of Imai et al. (2010), counterfactual mediator and outcome values were simulated based on fitted regression models: the mediator model regressed the mediator on intervention and covariates, while the outcome model regressed the outcome on the mediator, intervention, and covariates [35]. All confidence intervals (CIs) were derived from 2000 parametric bootstrap replications [35]. All analyses were performed in R software (version 4.5.2) using the ‘mediation’ package.

#### Sensitivity Analyses

To examine robustness, we conducted a sensitivity analysis to assess the degree to which the estimated ACME would be affected by potential unmeasured confounding in the mediator–outcome relationship, as proposed by Imai et al. (2010) [35]. For the linear regression models underlying the mediation analyses, model adequacy was assessed using standard diagnostics (residual-versus-fitted and normal Q-Q plots, scale–location plots, and Cook’s distance), and model fit was summarized using R^2^, adjusted R^2^, and the residual standard error [36]. In exploratory analyses, we also examined the association between change in self-efficacy and change in disability and tested whether the relationship between change in self-efficacy and change in disability differed by treatment group in a linear regression model predicting change in RMDQ [36].

## 3. Results

### 3.1. Participant Characteristics

Table 1 presents the baseline characteristics and post-intervention outcomes for participants in the control and pain education groups (n = 46 each). Both groups were generally comparable at baseline in terms of age, gender distribution, BMI, smoking status, physical activity level, sleep duration, and baseline clinical measures. The mean age was similar (42.52 ± 13.32 years in the control group vs. 42.02 ± 8.24 in the pain education group), and most participants were female. Sleep duration was comparable at baseline (6.33 ± 1.73 h vs. 6.74 ± 1.32), and smoking prevalence was higher in the intervention group (26.1%) compared to the control group (8.7%).

At baseline, pain intensity scores were 5.98 ± 1.71 for the control group and 5.70 ± 1.66 for the pain education group. Following the intervention, the control group exhibited minimal change in pain intensity (5.57 ± 1.15), whereas the intervention group demonstrated a significant reduction (2.26 ± 0.88). In the control group, disability scores (RMDQ) exhibited a minor decrease (from 15.02 ± 4.78 to 14.00 ± 4.74), while the intervention group demonstrated a significant reduction (from 12.65 ± 4.71 to 5.80 ± 2.49).

Improvements were also observed in the emotional well-being assessed by WHO-5, increasing from 53.48 ± 17.53 to 70.13 ± 14.42 in the intervention group, while the control group changed only modestly (54.41 ± 18.56 to 56.39 ± 16.34). GSE followed a similar pattern, with greater post-intervention improvement in the pain education group (from 25.83 ± 5.61 to 28.85 ± 5.54) compared to the control group (from 25.78 ± 6.40 to 30.28 ± 5.84).

### 3.2. Mediation Analysis

#### 3.2.1. Causal Mediation Effects on Disability (RMDQ)

As shown in Table 2, causal mediation analysis revealed that the WHO-5 significantly mediated the effect of pain education on disability scores (ACME = −1.66, 95% CI [−2.8, −0.72], *p* < 0.001), accounting for approximately 23.6% of the total effect. Neither self-efficacy (ACME = 0.10, 95% CI [−0.15, 0.37], *p* = 0.41) nor pain intensity (ACME = −0.69, 95% CI [−2.67, 1.40], *p* = 0.51) showed significant indirect effects. The mediation model, including multiple mediators, did not yield significant indirect effects on disability for the physiotherapy versus physiotherapy and education model (Table S1). The ADE and total effect estimates corresponding to these mediation results are also reported in Table 2 to provide a complete decomposition of effects. In exploratory stratified mediation analyses, the indirect effect of pain education versus control on disability at 6 weeks via WHO-5 was consistently negative across sex, smoking status, and duration of LBP subgroups, with broadly overlapping 95% confidence intervals (Table S2).

The mediator and outcome regression models showed good fit, explaining 70.7% and 76.5% of the variance in WHO-5 and RMDQ scores at 6 weeks, respectively (adjusted R^2^ = 0.697 and 0.754), and diagnostic plots did not indicate major violations of linear regression assumptions or any highly influential observations (Table S3, Figure S1). Exploratory analyses examining the association between change in self-efficacy and change in disability, and testing whether this relationship differed between groups, did not provide evidence of a meaningful relationship or differential effect between groups. Full results are presented in Table S4.

#### 3.2.2. Causal Mediation Effects on Pain Intensity (VAS)

As shown in Table 3, none of the tested mediators significantly mediated the effect of pain education on post-intervention pain intensity. The indirect effects (ACME) through the General Self-Efficacy Scale, WHO-5 Well-being Index, and Roland-Morris Disability Questionnaire were all non-significant (ACME = 0.05, 95% CI [−0.02, 0.16], *p* = 0.254; ACME = −0.18, 95% CI [−0.47, 0.1], *p* = 0.226; and ACME = −0.13, 95% CI [−0.54, 0.29], *p* = 0.580, respectively). The proportion of the total effect mediated ranged from −1.4% to 5.7%. The mediation model, including multiple mediators, did not yield significant indirect effects on pain intensity for the physiotherapy versus physiotherapy and education model (Table S5).

### 3.3. Sensitivity Analyses

**Figure 1:**
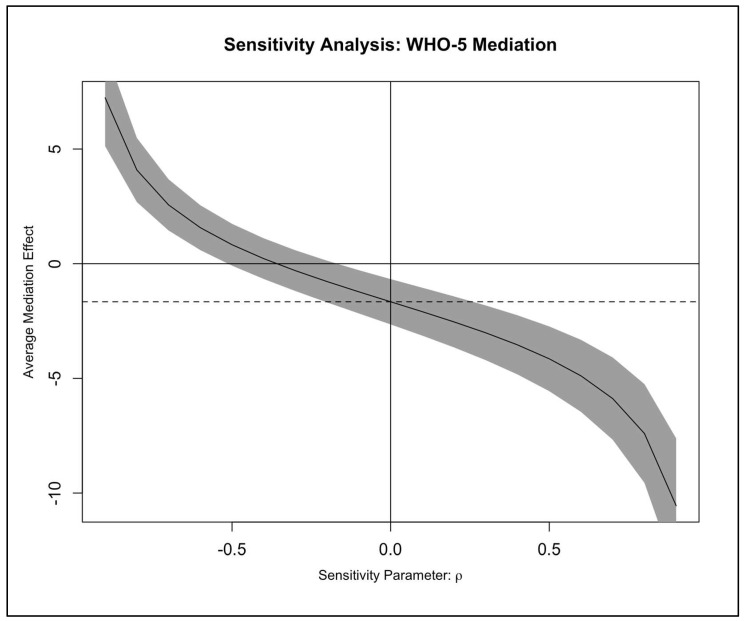
Sensitivity analysis for the confounding assumption in the mediation model for WHO-5. The solid curve represents the estimated average causal mediation effect (ACME) of the WHO-5 Well-being Index on disability (RMDQ) as a function of the sensitivity parameter (ρ). The shaded area indicates the 95% confidence interval, the horizontal dotted line denotes the null indirect effect (ACME = 0), and the vertical line at ρ = 0 represents the assumption of no residual correlation between the mediator and outcome models.

Sensitivity analysis indicated that the mediating effect of psychological well-being (WHO-5) on disability was robust to a meaningful degree of unmeasured mediator–outcome confounding. Although psychological variables such as depression, catastrophizing, or fear-avoidance were not measured in the original trial, the sensitivity curve demonstrates that the ACME would remain statistically significant unless the residual correlation (ρ) between the mediator and outcome models exceeded approximately −0.40 (equivalent to a shared variance of 16%). Confidence intervals for the ACME remained above zero across most of the plausible range of ρ values, suggesting that unmeasured psychological confounders would need to exert a strong and unlikely degree of correlated influence to fully explain away the mediation effect. The sensitivity curve illustrating this robustness is presented in Figure 1, with detailed numerical estimates included in Table S6.

## 4. Discussion

### 4.1. Summary of Findings

This secondary analysis of an RCT investigated whether the impacts of a pain education program on pain and disability were facilitated by self-efficacy and psychological well-being. The findings offer partial support for the proposed hypothesis of the mediation pathways. Pain education, when provided together with physiotherapy, substantially reduced pain and disability in comparison to physiotherapy alone, as evidenced in the initial RCT [23]. Mediation analyses indicated that improvements in emotional well-being were associated with approximately 24% of the overall difference in disability between groups. On the other hand, self-efficacy did not have a significant effect on disability, and neither self-efficacy nor psychological well-being had an effect on the intervention’s effect on pain intensity. Because the mediator and outcome were measured concurrently at the 6-week follow-up, the temporal ordering required for definitive causal mediation cannot be confirmed.

### 4.2. The Mediating Role of Psychological Well-Being in the Effect of Pain Education on Disability

Our results indicate that improvements in emotional well-being were associated with lower disability scores and partially explained the differences observed between groups. This suggests that improvements in the general quality of life could be partly responsible for the reduction in disability observed in the intervention group. On the other hand, the observed treatment effect was not significantly explained by other mediators that were assessed in this study, such as pain intensity and pain self-efficacy.

These results are consistent with a growing body of research highlighting the importance of psychological health in musculoskeletal rehabilitation. Psychological interventions improve physical function and quality of life for people with chronic LBP, particularly when paired with physiotherapy [37]. CBT and mindfulness-based stress reduction (MBSR) have also shown promise in improving psychological and functional outcomes and reducing pain in this population [19,20]. Additionally, a comprehensive analysis by O’Keeffe et al. [38] emphasized that pain-related disability can be considerably reduced by addressing maladaptive pain-related thoughts, feelings, and behavioral tendencies.

The study’s findings regarding the mediating role of psychological well-being align with the biopsychosocial model of chronic pain. This model recognizes that social and psychological factors, in addition to biological influences, have a substantial impact on disability [5]. The WHO-5 is an internationally recognized tool for assessing well-being and has been validated among clinical groups, including those with chronic pain [12]. It might also cover broader aspects of mental health that are not just connected to pain. In line with evidence showing that psychosocial constructs often show a stronger correlation with disability than with pain severity, this could help explain why well-being mediated disability but not pain intensity [9]. These findings suggest that interventions that support psychological well-being may enhance functional outcomes and could serve as a valuable addition to traditional rehabilitation approaches. Integrating mental health components into pain education and rehabilitation programs may be a vital approach to improve long-term outcomes for those with LBP. The absence of mediation through pain intensity further suggests that psychological well-being may influence disability via affective-motivational or attentional pathways, rather than through direct modulation of nociceptive processing [39]. However, because well-being and disability were measured at the same time point in this study, alternative temporal sequences cannot be excluded, and the proposed pathway should be regarded as preliminary.

### 4.3. Self-Efficacy as a Non-Significant Mediator

Although self-efficacy is widely acknowledged as a key predictor of outcomes in chronic LBP, our mediation analysis did not support its role in explaining the benefits of pain education on either pain or disability. These null mediation findings are consistent with exploratory analyses in the present dataset, which showed only a very weak, non-significant association between change in self-efficacy and change in disability and no evidence that this association differed between intervention and control groups (Table S4). This contrasts with prior evidence. For example, van Hooff et al. demonstrated that post-treatment self-efficacy was a stronger predictor of functional improvements than traditional constructs such as fear-avoidance or catastrophizing [40]. Similarly, Ryum and Stiles reported that increases in self-efficacy significantly mediated the relationship between changes in pain intensity and disability in the treatment of LBP [15]. Meta-analyses also highlight self-efficacy as a robust determinant of pain-related disability across musculoskeletal conditions [7].

Several explanations may account for the absence of mediation effects in the present study. First, the short intervention duration (six weeks) may not have been sufficient to produce measurable improvements in self-efficacy. A recent meta-analysis indicated that exercise or combined physiotherapy programs typically yield only small improvements in self-efficacy over longer periods of 4–6 months [7]. Second, the content of the pain education program primarily focused on neurophysiological understanding and promoting psychological well-being, rather than mastery-based or behavioral strategies such as graded exposure, goal setting, or cognitive-behavioral therapy techniques that directly enhance self-efficacy. Finally, although baseline self-efficacy scores were in the moderate range, suggesting scope for improvement, the intervention may not have sufficiently targeted the mechanisms necessary to shift this construct, thereby limiting its potential role as a mediator. It is also possible that the content of the pain education intervention targeted emotional appraisal more directly than behavioral confidence, and that the use of a global (non-pain-specific) self-efficacy measure may have reduced sensitivity to detect change in this context.

### 4.4. Implications and Future Directions

The findings from this study have both clinical and research implications. Clinically, the results highlight the importance of addressing psychological well-being in rehabilitation programs for individuals with chronic LBP. The observation that improvements in well-being, but not self-efficacy, mediated reductions in disability suggests that interventions that foster positive mental health and resilience may be particularly effective in improving functional outcomes. Routine use of brief, validated tools such as the WHO-5 could help clinicians identify patients with low emotional well-being who may benefit most from pain education and complementary psychosocial support [12]. Integrating strategies that actively target mental well-being, such as mindfulness-based interventions, positive psychology techniques, and stress management, in physiotherapy and pain education programs may therefore optimize long-term outcomes [41,42]. These implications should therefore be interpreted cautiously and primarily as guidance for future mechanistic and clinical trials, rather than as definitive evidence. These exploratory findings are consistent with a recent umbrella review showing that pain neuroscience education combined with exercise or physiotherapy yields small-to-moderate improvements in pain and disability in chronic LBP, while the mechanisms underlying these benefits still require further clarification [14].

An alternative explanation for the observed pattern is moderation rather than mediation, for example, that individuals with higher baseline well-being or self-efficacy may derive greater benefit from pain education. Although exploratory analyses did not reveal significant interaction effects, this remains a plausible conceptual model and warrants examination in future trials with repeated mediator assessments.

From a research perspective, the findings underscore the need to broaden the scope of mediation analyses in chronic LBP. While this study focused on psychological well-being and self-efficacy, other psychosocial mechanisms, including fear-avoidance, catastrophizing, stress, and mood disturbance, may also contribute significantly to the effects of education and rehabilitation programs [10,34]. In addition, future studies should investigate physiological and behavioral pathways, such as nociceptive modulation and physical activity, to capture a more comprehensive understanding of treatment mechanisms [43]. Longer follow-up periods are also essential to determine whether improvements in psychological well-being translate into sustained functional benefits and whether self-efficacy requires greater time and targeted intervention strategies to emerge as a significant mediator [44,45].

### 4.5. Strengths and Limitations

This study has several notable strengths. First, it was based on a rigorously conducted RCT, which enhances internal validity by minimizing selection bias and treatment–mediator confounding. The use of a counterfactual causal mediation framework allowed for the decomposition of total effects into direct and indirect components, providing a nuanced understanding of how pain education exerts its influence on clinical outcomes. To further ensure robustness, confidence intervals were generated using bootstrap resampling, and sensitivity analyses were conducted, demonstrating that the mediating role of psychological well-being was relatively stable even under moderate levels of unmeasured confounding. In addition, supplementary sensitivity models that included baseline smoking status, BMI, and duration of low back pain yielded ACME estimates nearly identical to the primary model, further supporting the robustness of the mediating effect of psychological well-being. These methodological features strengthen confidence in the validity of the observed findings.

Despite the strengths of this study, several limitations should be noted. First, because this was a secondary analysis, the original trial was not specifically powered to detect mediation effects, which may have limited our ability to identify smaller indirect pathways. This limitation is even more pronounced for the exploratory subgroup mediation analyses (Table S2), which were based on smaller strata and multiple comparisons. Accordingly, these subgroup results were presented primarily to assess consistency of the mediation pattern across key clinical characteristics, rather than to support formal hypothesis testing, and the associated *p*-values should be regarded as descriptive. Second, the follow-up period was relatively short (six weeks), making it difficult to draw conclusions about the durability of the intervention’s effects and potentially explaining why self-efficacy did not emerge as a mediator. Third, this study focused on only two mediators (psychological well-being and self-efficacy) while other psychosocial factors, such as fear-avoidance, catastrophizing, stress, or anxiety, were not measured and may also play important roles in treatment mechanisms. Fourth, mediators and outcomes were collected at the same post-intervention time point. Although this study adjusted for baseline levels, this limits the ability to establish temporal ordering, and the mediation findings should be interpreted with caution. Finally, as with any causal mediation analysis, the sequential ignorability assumption cannot be fully verified, even though the sensitivity analyses suggest that a substantial degree of unmeasured confounding would be required to eliminate the observed mediation effect.

## 5. Conclusions

This secondary analysis found that improvements in psychological well-being were associated with reductions in disability following pain education, whereas self-efficacy did not appear to explain treatment effects. These mediation findings should be interpreted cautiously, as mediators and outcomes were assessed concurrently, and the short intervention period limits conclusions about temporal ordering or durability. Nonetheless, the results suggest that psychological well-being may play a meaningful role in functional improvement and highlight the value of incorporating well-being-oriented content into rehabilitation programs.

## Figures and Tables

**Table 1:** Baseline and Post-Intervention Characteristics of Participants in the Pain Education and Control Groups of 92 participants.

Variable	Level	Control (n = 46)	Pain Education (n = 46)	Control (6 Weeks Post-Intervention) (n = 46)	Pain Education (6 Weeks Post-Intervention) (n = 46)
Age (years)		42.52 (13.32)	42.02 (8.24)		
Gender	Female	35 (76.1)	25 (54.3)		
	Male	11 (23.9)	21 (45.7)		
BMI (kg/m^2^)		26.96 (4.08)	27.76 (4.28)		
Duration of LBP (months)		35.85 (50.37)	20.93 (34.33)		
Level of activity	Light	14 (30.4)	17 (37.0)		
	Moderate	23 (50.0)	20 (43.5)		
	Very active	9 (19.6)	9 (19.6)		
Smoker	No	42 (91.3)	34 (73.9)		
	Yes	4 (8.7)	12 (26.1)		
Hours of sleep		6.33 (1.73)	6.74 (1.32)		
VAS (0–10)		5.98 (1.71)	5.70 (1.66)	5.57 (1.15)	2.26 (0.88)
RMDQ (0–24)		15.02 (4.78)	12.65 (4.71)	14.00 (4.74)	5.80 (2.49)
WHO-5 (0–100)		54.41 (18.56)	53.48 (17.53)	56.39 (16.34)	70.13 (14.42)
GSE (10–40)		25.78 (6.40)	25.83 (5.61)	30.28 (5.84)	28.85 (5.54)

Values are presented as mean (SD) or frequency (%), as appropriate. Abbreviations: BMI = Body Mass Index; LBP = Low Back Pain; VAS = Visual Analog Scale for pain intensity; RMDQ = Roland-Morris Disability Questionnaire; WHO-5 = World Health Organization-5 Well-Being Index; GSE = General self-efficacy scale.

**Table 2:** Estimated total, direct (ADE), and indirect (ACME) effects of pain education versus control on Roland-Morris Disability Questionnaire scores at 6 weeks, with effect estimates and 95% confidence intervals for each potential mediator.

Mediator	Path a ^1^	Path b ^2^	Total Effect ^3^ [95% CI]	Direct Effect (ADE) [95% CI]	Indirect Effect (ACME) [95% CI]	ACME *p*-Value	% Mediated
GSE	−1.66 [−3.48, 0.16]	−0.06 [−0.21, 0.09]	−7.02 [−8.37, −5.66]	−7.11 [−8.5, −5.68]	0.1 [−0.15, 0.38]	0.410	−1.4
WHO-5	14.49 [10.53, 18.45]	−0.11 [−0.18, −0.05]	−7.02 [−8.37, −5.69]	−5.37 [−7.13, −3.67]	−1.66 [−2.8, −0.72]	0.001	23.6
VAS	−3.23 [−3.6, −2.86]	0.21 [−0.51, 0.94]	−7.01 [−8.4, −5.68]	−6.32 [−8.67, −4.09]	−0.69 [−2.56, 1.39]	0.508	9.9

Abbreviations: WHO-5 = World Health Organization-5 Well-Being Index; ADE = Average Direct Effect; ACME = Average Causal Mediation Effect; CI = Confidence Interval; VAS = Visual Analog Scale for pain intensity. ^1^ Path a is intervention–mediator estimates in units of the mediator scale. ^2^ Path b is mediator–outcome estimates in units of the outcome scale. ^3^ Total effect estimates are presented in terms of back-related disability on the RMDQ scale. All estimates are presented as mean differences between pain education and the control group and their 95% confidence interval. All estimates are presented as mean differences between pain education and the control group, with 95% confidence intervals.

**Table 3:** Estimated total, direct (ADE), and indirect (ACME) effects of pain education versus control on pain intensity at 6 weeks, with effect estimates and 95% confidence intervals for each potential mediator.

Mediator	Path a ^1^	Path b ^2^	Total Effect ^3^ [95% CI]	Direct Effect (ADE) [95% CI]	Indirect Effect (ACME) [95% CI]	ACME *p*-Value	% Mediated
GSE	−1.35 [−3.11, 0.4]	−0.03 [−0.08, 0.01]	−3.21 [−3.57, −2.86]	−3.26 [−3.61, −2.92]	0.05 [−0.02, 0.16]	0.254	−1.4
WHO-5	14.32 [10.47, 18.17]	−0.01 [−0.03, 0.01]	−3.21 [−3.57, −2.85]	−3.02 [−3.47, −2.58]	−0.18 [−0.47, 0.1]	0.226	5.7
RMDQ	−7.01 [−8.27, −5.74]	0.02 [−0.04, 0.08]	−3.23 [−3.59, −2.88]	−3.1 [−3.59, −2.58]	−0.13 [−0.54, 0.29]	0.580	4.0

Abbreviations: WHO-5 = World Health Organization-5 Well-Being Index; ADE = Average Direct Effect; ACME = Average Causal Mediation Effect; CI = Confidence Interval; RMDQ = Roland-Morris Disability Questionnaire. ^1^ Path a is intervention–mediator estimates in units of the mediator scale. ^2^ Path b is mediator–outcome estimates in units of the outcome scale. ^3^ Total effect estimates are presented in terms of pain intensity on the VAS scale. All estimates are presented as mean differences between the pain education and control group and their 95% confidence interval.

## Data Availability

This is a secondary analysis of a published randomized control trial. The data is available publicly. The R code used to perform the mediation analyses is available from the author upon reasonable request.

## Supplementary Materials

The following supporting information can be downloaded at https://www.mdpi.com/article/10.3390/jcm15010348/s1. Table S1: Decomposition of the total effect of physical therapy versus physical therapy and education on disability score assessd by RMDQ using combined multiple mediator model; Table S2: Stratified causal mediation analyses of the effect of pain education plus physiotherapy versus physiotherapy alone on disability (RMDQ) at 6 weeks via WHO-5, by sex, smoking status, and duration of low back pain; Table S3: Model fit indices for the mediator and outcome regression models used in the primary causal mediation analysis; Table S4: Exploratory analyses of the association between self-efficacy and disability; Table S5: Decomposition of the total effect of physical therapy versus physical therapy and education on disability score assessd by RMDQ using combined multiple mediator model; Table S6: Sensitivity Analysis of the Average Causal Mediation Effect (ACME) for the WHO-5 Mediator on Disability Outcomes; Figure S1: Diagnostic plots for the linear regression models underlying the primary mediation analysis. The top row shows the mediator model (WHO-5 at 6 weeks); the bottom row shows the outcome model (RMDQ at 6 weeks). Residual-versus-fitted, normal Q-Q, scale-location, and Cook’s distance plots did not indicate major violations of linear regression assumptions or any highly influential observations.
