# Supplementary material for: Causal Mediation Analysis of the Effects of Pain Education on Disability and Pain Intensity in Individuals with Chronic Low Back Pain
Source: J Clin Med. 2026 Jan 2;15(1):348. doi: 10.3390/jcm15010348 (PMC12787205; doi:10.3390/jcm15010348)
Supplement: Supplementary file 1 [file jcm-15-00348-s001.zip › jcm-3934074-supplementary.pdf]

Supplementary files:

Table S1. Decomposition of the total effect of physical therapy versus physical therapy and education on disability score assessd by RMDQ using combined multiple mediator model.

| **Mediator** | **ACME** | **ACME 95% CI** | **ACME p-value** | **ADE** | **ADE 95% CI** | **Total Effect** | **Total Effect 95% CI** |
|---|---|---|---|---|---|---|---|
| GSE | 0.05 | [−0.23, 0.34] | 0.716 | −5.43 | [−8.14, −2.73] | −5.38 | [−7.99, −2.74] |
| WHO−5 | −1.68 | [−2.85, −0.67] | 0 | −5.43 | [−7.97, −2.54] | −7.11 | [−9.59, −4.43] |
| VAS | 0.00 | [−2.06, 2.11] | 0.992 | −5.43 | [−8.02, −2.53] | −5.44 | [−7.13, −3.72] |
| Total Indirect | −1.63 | | | −5.43 | | −7.06 | |
| % Mediated | 23.10 | | | | | | |

Abberiviations: WHO−5 = World Health Organization−5 Well−Being Index; ADE = Average Direct Effect; ACME = Average Causal Mediation Effect; CI = Confidence Interval; VAS = Visual Analogue Scale for pain intensity.

Table S2. Stratified causal mediation analyses of the effect of pain education plus physiotherapy versus physiotherapy alone on disability (RMDQ) at 6 weeks via WHO−5, by sex, smoking status, and duration of low back pain.

| **Subgroup variable (level)** | **N** | **Total Effect [1] [95% CI]** | **Direct Effect (ADE) [95% CI]** | **Indirect Effect (ACME) [95% CI]** | **ACME p−value** | **% Mediated** |
|---|---|---|---|---|---|---|
| Sex (Female) | 60 | −7.19 [−8.84, −5.59] | −5.37 [−7.32, −3.58] | −1.82 [−3.28, −0.49] | 0.006 | 25.3 |
| Sex (Male) | 32 | −5.84 [−8.62, −2.70] | −4.98 [−8.38, −1.25] | −0.86 [−2.57, 0.30] | 0.170 | 14.7 |
| Smoking status (Non−smoker) | 76 | −7.22 [−8.82, −5.83] | −5.54 [−7.45, −3.73] | −1.68 [−3.06, −0.48] | 0.006 | 23.3 |
| Smoking status (smoker) | 16 | | Not estimated* | | | |
| Duration of low back pain (<12 months) | 56 | −7.32 [−9.60, −5.35] | −5.50 [−8.24, −2.99] | −1.82 [−3.24, −0.57] | 0.003 | 24.9 |
| Duration of low back pain (12–36 months) | 21 | −7.79 [−10.48, −4.64] | −5.04 [−7.92, −1.42] | −2.75 [−6.40, −0.04] | 0.045 | 35.3 |
| Duration of low back pain (>36 months) | 15 | | Not estimated* | | | |

ADE = Average Direct Effect; ACME = Average Causal Mediation Effect; CI = Confidence Interval.

[1] Total effect estimates are presented in terms of back−related disability on the RMDQ scale. All estimates are presented as mean differences between pain education versus control group and their 95% confidence interval.

All estimates are presented as mean differences between pain education and control, with 95% confidence intervals.

* Formal mediation models were only estimated for strata with at least 20 participants, and variables with fewer participants are reported as "Not estimated".

Subgroup mediation analyses are exploratory and were not powered for subgroup−specific effects; p−values are unadjusted for multiple comparisons and should be interpreted as descriptive.

Table S3: Model fit indices for the mediator and outcome regression models used in the primary causal mediation analysis.

| Model | Outcome | N | $R^2$ | Adjusted $R^2$ | Residual SE |
|---|---|---|---|---|---|
| Mediator model | WHO–5 at 6 weeks | 92 | 70.7% | 69.7% | 9.26 |
| Outcome model | RMDQ at 6 weeks | 92 | 76.5% | 75.4% | 2.77 |

Model fit statistics are based on the linear regression models used in the primary mediation analysis (WHO–5 at 6 weeks as mediator and RMDQ at 6 weeks as outcome).

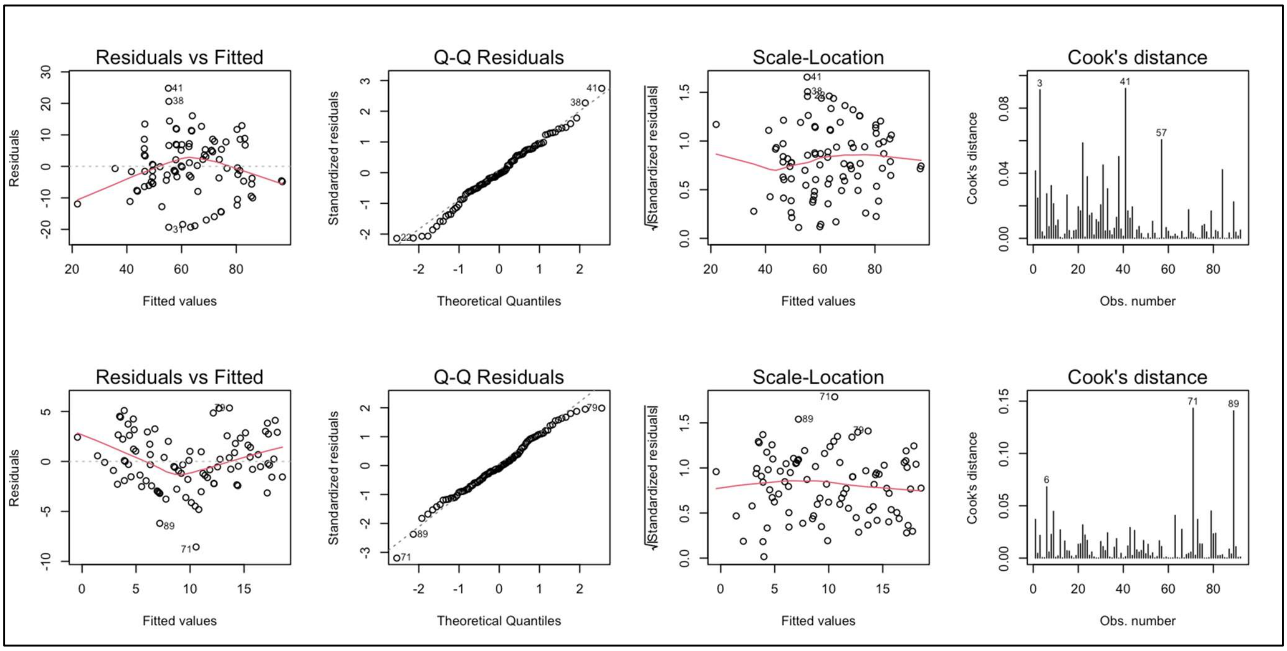


Figure S1. Diagnostic plots for the linear regression models underlying the primary mediation analysis. The top row shows the mediator model (WHO–5 at 6 weeks); the bottom row shows the outcome model (RMDQ at 6 weeks). Residual–versus–fitted, normal Q–Q, scale–location, and Cook’s distance plots did not indicate major violations of linear regression assumptions or any highly influential observations.

.

Table S4: Exploratory analyses related to self−efficacy.

To further examine the null mediation findings for self−efficacy, we explored its association with disability change. Across the whole sample, change in self−efficacy showed only a very weak, non−significant correlation with change in RMDQ scores (r = 0.09, 95% CI [−0.12, 0.29], p = 0.39). In a linear regression model predicting change in disability, the main effect of change in self−efficacy was small and non−significant (β = −0.09, 95% CI [−0.29, 0.11], p = 0.38). There was also no evidence that the relationship between change in self−efficacy and change in disability differed between intervention and control groups (β = 0.06, 95% CI [−0.20, 0.32], p = 0.66).

Table S4: Exploratory analyses of the association between self−efficacy and disability.

| Analysis / model | Outcome | Predictor | Effect estimate | 95% CI | p−value |
|---|---|---|---|---|---|
| Pearson correlation [1] | RMDQ (change) | GSE (change) | r = 0.09 | [−0.12]–[0.29] | 0.39 |
| Linear regression (change model–main effect) [2] | RMDQ (change) | GSE (change) | β = −0.09 | [−0.29]–[0.11] | 0.38 |
| Linear regression (change model)–Group × GSE [3] | RMDQ (change) | Group × GSE (change) | β = 0.06 | [−0.20]–[0.32] | 0.66 |

RMDQ = Roland−Morris Disability Questionnaire; GSE= General self−efficacy scale.
[1] Pearson correlation between change in self−efficacy and change in disability.
[2] Linear regression model predicting change in disability from change in self−efficacy.
[3] Linear regression model predicting change in disability including an interaction term between treatment group and change in self−efficacy; the coefficient shown represents the difference in the self−efficacy−disability slope between intervention and control groups.
Overall, there was no evidence that changes in self−efficacy were strongly associated with changes in disability or that this relationship differed between treatment groups.

Table S5: Decomposition of the total effect of physical therapy versus physical therapy and education on disability score assessd by RMDQ using combined multiple mediator model

| Mediator | ACME | ACME 95% CI | ACME p−value | ADE | ADE 95% CI | Total Effect | Total Effect 95% CI |
|---|---|---|---|---|---|---|---|
| GSE | 0.05 | [−0.02, 0.17] | 0.252 | −3.12 | [−3.64, −2.59] | −3.07 | [−3.58, −2.55] |
| WHO−5 | −0.17 | [−0.49, 0.15] | 0.275 | −3.12 | [−3.63, −2.57] | −3.29 | [−3.83, −2.71] |
| RMDQ | 0.00 | [−0.45, 0.47] | 0.994 | −3.12 | [−3.63, −2.59] | −3.12 | [−3.6, −2.68] |
| Total Indirect | −0.12 | | | −3.12 | | −3.24 | |
| % Mediated | 3.70 | | | | | | |

Abberiviations: WHO−5 = World Health Organization−5 Well−Being Index; ADE = Average Direct Effect; ACME = Average Causal Mediation Effect; CI = Confidence Interval; VAS = Visual Analogue Scale for pain intensity.

Table S6: Sensitivity Analysis of the Average Causal Mediation Effect (ACME) for the WHO–5 Mediator on Disability Outcomes

| **Rho (ϱ)** | **ACME** | **95% CI Lower** | **95% CI Upper** | **$R^2$_M × $R^2$_Y** |
|---|---|---|---|---|
| −0.5 | 0.8273 | −0.0846 | 1.7391 | 0.25 |
| −0.4 | 0.2203 | −0.6667 | 1.1072 | 0.16 |
| −0.3 | −0.305 | −1.1938 | 0.5837 | 0.09 |
| −0.2 | −0.7804 | −1.6894 | 0.1285 | 0.04 |

Abberviations: ϱ (Rho): Hypothetical correlation between the error terms of the mediator and outcome models; represents the degree of unmeasured confounding; ACME: Average Causal Mediation Effect; 95% CI Lower / Upper: The lower and upper bounds of the 95% confidence interval for the ACME; $R^2$_M × $R^2$_Y: Product of the proportions of variance explained in the mediator and outcome models by an unmeasured confounder; a measure of how strongly such a confounder would need to influence both to explain away the observed mediation effect.
